# Computational methods of EEG signals analysis for Alzheimer’s disease classification

**DOI:** 10.1038/s41598-023-32664-8

**Published:** 2023-05-20

**Authors:** Mário L. Vicchietti, Fernando M. Ramos, Luiz E. Betting, Andriana S. L. O. Campanharo

**Affiliations:** 1 Department of Biodiversity and Biostatistics, Institute of Biosciences, São Paulo State University, Botucatu, 18618-689 Brazil; 2 National Institute for Space Research, Earth System Science Center, São José dos Campos, 12227-010 Brazil; 3 Department of Neurology, Psychology and Psychiatry, Botucatu Medical School, São Paulo State University, Botucatu, 18618-687 Brazil

**Keywords:** Alzheimer's disease, Biomedical engineering, Complex networks

## Abstract

Computational analysis of electroencephalographic (EEG) signals have shown promising results in detecting brain disorders, such as Alzheimer’s disease (AD). AD is a progressive neurological illness that causes neuron cells degeneration, resulting in cognitive impairment. While there is no cure for AD, early diagnosis is critical to improving the quality of life of affected individuals. Here, we apply six computational time-series analysis methods (wavelet coherence, fractal dimension, quadratic entropy, wavelet energy, quantile graphs and visibility graphs) to EEG records from 160 AD patients and 24 healthy controls. Results from raw and wavelet-filtered (alpha, beta, theta and delta bands) EEG signals show that some of the time-series analysis methods tested here, such as wavelet coherence and quantile graphs, can robustly discriminate between AD patients from elderly healthy subjects. They represent a promising non-invasive and low-cost approach to the AD detection in elderly patients.

## Introduction

Alzheimer’s disease (AD) is a neurological disorder of the brain that causes neuronal cells degeneration. Despite the fact that AD still does not have a clear established origin, it is known that patients with AD suffer from cognitive loss, including memory and space-time perception. Currently, AD is considered the most common form of dementia, affecting nearly 50% of people over age 85^1,2^. Since AD is a progressive disease, its symptoms range from mild ones, that do not significantly affect patients’ daily lives, to severe ones, that lead patients to complete cognitive deterioration, dependence, and finally death^3,4^.

AD may be divided into three stages. In the initial stage, the AD phenotype follows a pattern of a Mild Cognitive Impairment (MCI)^5^, usually characterized by loss of recent memory, indicating the compromising of the medial temporal lobe structures, including the hippocampus. Annually, 10–15% of patients diagnosed with MCI progress to AD dementia^6^. MCI occurs during the translational period between healthy and pathological brain, but not all MCI patients will evolve to AD. That’s why it is important to identify biomarkers capable of predicting the transition from MCI to AD^5,7^. In the intermediary stage, the neurodegenerative process spreads outside the temporal lobe, causing diffuse cognitive dysfunction and affecting daily living activities. In the advanced stage, the individuals are totally dependent on caregivers and are usually restricted to bed^8,9^.

While there is no cure for AD, early diagnosis is critical to improve the selection and management of therapies (pharmacological and non-pharmacological), which leads to a better quality of life for the affected individuals, their relatives, and caregivers^10^; see ref.^6^ for a recent review on AD early diagnosis. In the initial stage, a distinction between AD symptoms and normal aging may be challenging. For that reason, examination of the brain tissue removed by biopsy or necropsy is still the gold standard for a definitive and precise AD diagnosis, and non-invasive techniques that can be used in clinical practice are still under investigation^10^. Psychological tests, brain imaging, and neuronal signal recording are examples of tests used for that purpose, which may help specialists discard other causes of dementia and diagnose AD^11–13^. Although recent works have proposed to combine two or more of the mentioned diagnosis tests, it is not clear how biomarkers can be correlated and, as in so many cases, these tests are unfeasible by their cost^14,15^. In this context, electroencephalography (EEG) has gained attention from the scientific community, since it is an inexpensive, widely available, non-invasive, and mobile technique^16,17^.

Basically, an EEG records the electrical potential generated by the physiological activities of the neurons^18,19^. The electric currents generated by depolarization of the cells’ membranes can create waves that are detected by scalp electrodes, originating signals that represent the neurons’ firing activity^20,21^. EEG signals cannot capture the activities of a single neuron, but a summation of synchronous firing of a group of cortical cells is usually recorded. Visual inspection of an EEG signal is often done by a trained neurologist, and even in this case, its analysis can be a difficult task due to the artifacts and noise contained in the signal^22,23^. Moreover, the multiple neural activities show complex and non-linear dynamics, which requires the use of sophisticated methods for measuring such behavior to reach higher sensitivity than in visual analysis^6,24^.

Many research groups have attempted to use computational analysis of EEG signals, and many different methods have shown promising results in detecting brain diseases^25–29^. Methods derived from information theory, time-frequency decomposition, and graph theory are examples of computational tools used in distinguishing healthy from unhealthy subjects^30–36^. Several studies in the literature have shown that AD causes slowing of the EEG rhythms, reduction in EEG complexity, and changes in synchrony among brain regions (see^22^ and references there in). In this sense, the aim of this study is to apply different methods of detecting AD through EEG signals and investigate the properties of the signals that distinguish the groups of patients. In particular, the sensitivity for discriminating AD patients from healthy elderly subjects was evaluated in terms of the area under the ROC curve and the ANOVA test. In a previous study, it was reported that a high accuracy in detecting AD through EEG was achieved using a small compilation of signals of 24 AD patients and 24 healthy controls^37^. The database in this paper was extended to 160 AD patients, and different computational methods were applied in order to compare their performances and their computational cost.

Algorithm complexity is a topic that scientists, mostly in computer science, have been recently investigating. This term means the amount of time and the computational storage needed to execute an algorithm, and it is related to the number of steps and operations involved in its calculations. In this sense, several research groups have developed new algorithms for EEG analysis that are faster and less complex^38,39^. Since the processing time of an algorithm increases with the input size of data, the search for efficient algorithms is an important research topic concerning the use of large data sets. In terms of EEG exams, the signals can last minutes or even hours, resulting in a large amount of data and, thus, making the use of high-complexity algorithms not practical. Therefore, algorithms with lower complexity can be more efficient in clinical and scientific practice when investigating AD. This aspect, not addressed in our previous work^37^, is also investigated here.

## Data

The EEG database used in this study was provided by researchers at Florida State University. The EEG signals are divided into four groups: (A) 12 healthy elderly with eyes open by visually fixating; (B) 12 healthy elderly with eyes closed; (C) 80 probable AD patients with eyes open by visually fixating; and (D) 80 probable AD patients with eyes closed. The 160 probable AD patients were diagnosed through the National Institute of Neurological and Communicative Disorders and Stroke (NINCDS) and the Alzheimer’s Disease and Related Disorders Association (ADRDA), and Diagnostic and Statistical Manual of Mental Disorders (DSM)-III-R criteria^40^. Due to a lack of information regarding the severity of the disease, the patients in the study were not sub-grouped according to their level of cognitive impairment.

EEG segments of 8-s duration were recorded at a sampling frequency of 128 Hz from the 19 scalp electrodes (Fp1, Fp2, F3, F4, F7, F8, Fz, C3, C4, Cz, P3, P4, Pz, T3, T4, T5, T6, O1, O2), according to the international 10–20 system of electrode placement. The signals were previously band-limited to the range of 0.5–30 Hz and the movement artifacts were removed from all the recordings by an EEG technician^41^. The database was provided by Dr. Dennis Duke and made freely available by Vicchietti et al.^42^.

## Methods

### Data preprocessing

EEG signals contain electrical activities from the brain as well as artefacts from other sources such as muscles movements and physical interference from the equipment^43,44^. Therefore, the need for preprocessing those signals before analyzing them is crucial. Digital filtering has become the most popular method of rejecting unwanted information in certain EEG frequencies^45^. Discrete Fourier Transform (DFT) has showed itself as a powerful tool in developing digital filters, once it generates the spectrum of a given signal. The spectrum represents the original signal in the frequency domain and denotes the power of each frequency band present in it. Thus, it is possible to attenuate the effect of a given frequency band in the spectra and, through the Inverse Fourier Transform, to return to the time domain without that band^46^. Nevertheless, DFT has limitations when it is applied to non-stationary (e.g. EEG) signals^47^. Stationary signals are those whose statistical measures, such as mean, variance and covariance remain constant over time in any sample of the input data^48^. Many of the DFT limitations were overcome by the use of wavelets, which consist of mathematical functions that are able to decompose a signal into various time-frequency scales by convolution operations^49^. The employment of wavelets in a given signal decomposition generates coefficients that keep the signal information; therefore, these coefficients can be used to reconstruct the signal through the inverse operation^50^.

The partition of given EEG components is useful for many specialists since each known EEG rhythm is related to specific brain stages and can be used to investigate the action of drugs, stimuli, and diseases on neuronal dynamics^12^. In this sense, previous studies have shown the decrease of high-frequency and the increase of low-frequency components activity in AD patients’ signals^51^. Therefore, the Daubechies-4 wavelet^52^ was used to extract the four most used EEG frequency bands, i.e., beta (15–30 Hz), alpha (8–15 Hz), theta (4–8 Hz) and delta (0–4 Hz). It is important to emphasize that, in this study, Wavelet Transform was the only tool used in the preprocessing step.

### Feature extraction

We present six of the more well-known techniques used in the literature for the distinction of AD from healthy elderly patients through EEG signals. Each technique was evaluated in terms of its capacity to discriminate between the groups of subjects. For this purpose, the area under the ROC curve (AUC) and the *p* value from the ANOVA test were calculated. AUC is a largely used measure that represents the effectiveness of a given diagnostic marker. The estimation of the AUC value is done through the probabilities of reaching true positive and true negative rates in a two-group classification. In this sense, AUC assumes values from 0.5 (no apparent distinction) to 1.0 (perfect distinction) between the two groups^53^. On the other hand, ANOVA is a powerful statistical test that is posed by the null hypothesis that two or more samples come from the same population. In this sense, the *p* value from ANOVA measures the probability of not rejecting the null hypothesis in such a manner that the probability of there existing more than one population increases as the *p* value comes closer to 0^54^. Moreover, from the point of view of computational cost, the execution time as a function of the signal input size was calculated for each technique.

**Wavelet coherence** (${\mathscr {C}}$) Wavelets are special functions that satisfy certain mathematical requirements and are used to represent data or other functions by decomposing them into a series of coefficients. Wavelet algorithms process data at different scales or resolutions^55^. The Wavelet Transform (WT) is used to calculate the coefficients *C*(*a*, *b*) in the scale *a* and time *b* as follows:

1 $$\begin{aligned} C(a,b) = \frac{1}{\sqrt{a}}\int _{-\infty }^{\infty }f(t)\Psi \left( \frac{t-b}{a} \right) dt, \end{aligned}$$

where *f*(*t*) is a time series or a function and $\Psi$ is the wavelet function.

The coherence ${\mathscr {C}}$ between two time series *X* and *Y* can be calculated using the coefficients generated by a WT. This measure represents the agreement between *X* and *Y* in different frequency levels through the time domain^56^. It can be calculated as follows:

2 $$\begin{aligned} {\mathscr {C}}=\frac{C_{X}C_{Y}^{*}}{\sqrt{C_{X}^{2}}\sqrt{C_{Y}^{2}}}. \end{aligned}$$

**Fractal dimension** (${\mathscr {F}}$) In recent years, the study of nonlinear systems has made possible the creation of metrics capable of quantifying random properties in time series^57^. One of these metrics is the Fractal Dimension (FD), which measures the complexity and the similarity of a given signal with itself^58^. A fractal can be defined as a geometric structure in which its parts repeat the spatial patterns of its whole body. In this matter, the FD of a signal represents its degree of randomness, whereas totally random series present no pattern repetition and, as a consequence, the signal becomes more complex. Different algorithms were proposed to estimate the fractality of a signal^59,60^. In this paper, we applied the one proposed by Katz^60^. First, the total length *D* of a signal *X* with *T* points, defined as the sum of the distances between any two adjacent points, is computed as follows:

3 $$\begin{aligned} D = \sum _{i=1}^{T - 1} \sqrt{1+\left[ x(i) - x(i + 1)\right] ^2}. \end{aligned}$$

After, the greatest distance between the first point of *X* and all the other successive points, defined by *d*, is computed as follows:

4 $$\begin{aligned} d = \textrm{max}\left\{ \sqrt{(1 - i)^{2} + \left[ x(1) - x(i)\right] ^{2}} \quad \Vert \quad i=1,2,\ldots ,T \right\} . \end{aligned}$$

Finally, the fractal dimension ${\mathscr {F}}$ defined by Katz can be obtained by:

5 $$\begin{aligned} {\mathscr {F}} = \frac{ln\left[ T - 1\right] }{ln\left[ (dT - d)D^{-1}\right] }. \end{aligned}$$

**Quadratic entropy** (${\mathscr {Q}}$) The physical concept of entropy represents the degree of freedom in a dynamic system. In other words, entropy measures the degree of disorder of that system, following the idea that totally random systems are completely disordered^61^. For a given signal, the entropy quantifies its regularity, so that signals with high freedom degrees also have high entropy and become less regular^62^. The entropy of a signal *X* is calculated by finding matches between its $T-m+1$ partitions that have *m* adjacent points in each one. The difference between the scalar components of the partitions $X_{i}$ and $X_{j}$, with $i,j= 1,2, \ldots , N-m+1$, is defined as the distance $\textrm{d}$ between them^63,64^:

6 $$\begin{aligned} \textrm{d}\left[ X_{i},X_{j} \right] = \textrm{max}\{ |x(i+k)-x(j+k)| \}, \qquad k=0,\ldots ,m-1. \end{aligned}$$

Then, $B^{m}(r)$ is calculated as the probability that two partitions match for *m* points with tolerance *r*^65^:

7 $$\begin{aligned} B^{m}(r)=\frac{1}{T-m} \sum _{i=1}^{T-m} \frac{1}{T-m-1}B_{i}, \end{aligned}$$

where *r* denotes the tolerance level and $B_{i}$ denotes the sum of the quantities of *j* that satisfy 1 $\le j$ $\le T-m$ with $j \ne i$ for any $X^{m}(i)$. In other words, *j* is the number of times $\textrm{d}\left[ X_{m}(i),X_{m}(j) \right] \le r$ occurs. The same steps are repeated with the increment to $m+1$, originating the combinations $A_{i}$ and $A^{m}(r)$:

8 $$\begin{aligned} A^{m}(r)=\frac{1}{T-m+1} \sum _{i=1}^{T-m+1} \frac{1}{T-m}A_{i}. \end{aligned}$$

The entropy *E* is obtained by:

9 $$\begin{aligned} E=-ln\left[ \frac{A^{m}(r)}{B^{m}(r)} \right] . \end{aligned}$$

When calculated for short time series, the entropy is limited^66^. Furthermore, studies have shown that the variation of the parameter *r* is increased when the entropy is calculated in terms of the Quadratic Entropy (QE). This measure can be obtained by:

10 $$\begin{aligned} {\mathscr {Q}}=E+ln(2r). \end{aligned}$$

**Wavelet energy** (${\mathscr {E}}$) The energy of a frequency band represents the importance of that component to composing the time series^67^. The coefficients generated through Eq. 1 can be used to calculate the energy of their components^56^. The use of wavelets in decomposing a time series requires the number of levels *J*, which is chosen based on the frequency range to be analyzed^68^. When a time series is decomposed, it generates $J+1$ sets of coefficients, which correspond to *J* detail coefficients sets and 1 approximation coefficients set^69^. The relative Wavelet Energy (WE) ${\mathscr {E}}_{j}$ of band *j* is defined as follows:

11 $$\begin{aligned} {\mathscr {E}}_{j}=\frac{\sum _{i=1}^{m_{j}}C(j, i)^{2}}{\sum _{j=1}^{J+1}\sum _{i=1}^{m_{j}}C(j, i)^{2}}, \end{aligned}$$

where $m_{j}$ is the number of coefficients associated to the band *j*.

**Quantiles graphs** ($\Delta$) A time series analysis method, which converts a time series into a Quantile Graph (QG), has emerged recently from the concepts of complex networks^39,70^. A complex network $g=\left\{ N, L\right\}$ is defined as a group of *N* nodes connected by *L* edges. In this method, a time series is coarse-grained into *Q* quantiles ${q_1,\dots ,q_Q}$. Each quantile $q_{i}$ represents a network node $n_{i}$, and each weight $a_{ij}^k$ in the weighted directed adjacency matrix, denoted as $A_{k}$, is equal to the number of times a value in quantile $q_{i}$ at time *t* is followed by a point in quantile $q_{j}$ at time $t+k$. This method can create directed weighted networks with $N_{QG}=Q$ vertices.

Based on the adjacency matrix $A_{k}$ and the Markov transition matrix $W_{k}$, mathematical metrics can be used in order to quantify different features of the corresponding network’s topology. In previous studies, the mean jump length, $\Delta _k$, was successfully used to characterize quantile graphs^23,30,37,39,71^. It is defined as follows:

12 $$\begin{aligned} \Delta _k = \frac{1}{N}tr(SP^{\mathscr {T}}), \end{aligned}$$

where $W^T_k$ is the transpose of $W_k$, *P* is a $Q \times Q$ matrix with elements $p_{i,j}=|i-j|$, and *tr* is the trace operation.

**Visibility graphs** (${\mathscr {I}}$) Another time series analysis method based on the complex network theory that converts a time series into a Visibility Graph (VG)^72^ has recently emerged from complex networks concepts. In this method, each point of a time series *X* is represented by a node in the corresponding network. Two nodes, $n_{i}$ and $n_{j}$, are connected if their corresponding points (*i*, *x*(*i*)) and (*j*, *x*(*j*)) in the time series are “visible” to each other. In other words, if any existing point (*k*, *x*(*k*)) between them satisfies the relationship:

13 $$\begin{aligned} x(k) \le x(j)+(x(i)-x(j)) \frac{j-k}{j-i}. \end{aligned}$$

The VG method can produce undirected unweighted networks with $N_{VG}=T$ vertices each. In previous studies, the complexity index ${\mathscr {I}}$ was successfully used to characterize visibility graphs^52,73^. It is defined as follows:

14 $$\begin{aligned} {\mathscr {I}}=4c(1-c), \end{aligned}$$

with:

15 $$\begin{aligned} c=\frac{\lambda _{\textrm{max}}-2cos(\pi /(N+1))}{N-1-2cos(\pi /(N+1))}, \end{aligned}$$

where $\lambda _{\textrm{max}}$ is the largest eigenvalue of the corresponding adjacency matrix.

## Results

We applied the previously described methods to the problem of discriminating normal controls from patients with AD, based on the 19 EEG channels available. For all channels, we calculated ${\mathscr {C}}$, ${\mathscr {F}}$, ${\mathscr {Q}}$, ${\mathscr {E}}$, $\Delta$, and ${\mathscr {I}}$ for the groups A, B, C, and D.

${\mathscr {C}}$ was calculated for all the possible combinations of a given electrode with the others. ${\mathscr {F}}$ and ${\mathscr {Q}}$ were calculated using $T=1,024$. ${\mathscr {Q}}$ was calculated using $r=0.05,\,0.10,\,0.15,\,\ldots ,\, 1.00$ with $m=1$ and $m=2$^63^. $\Delta$ was calculated using $N_{QG}=Q=2(1,024)^{1/3}\approx 20$ and $k=1,\,2,\,3,\,\ldots ,\,25$^37^. ${\mathscr {I}}$ was calculated using $N_{VG}=T=1,024$. For all the methods, the parameters were chosen in such a way to obtain the lowest *p* value of the ANOVA test.

**Figure 1:**
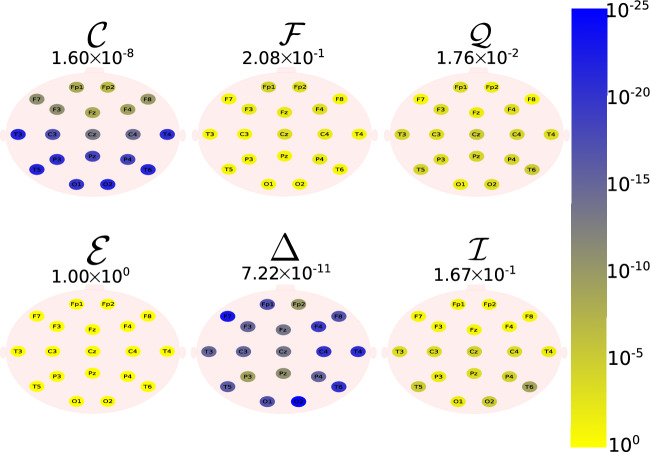
The location on the scalp of the 19 EEG original signal channels (groups A vs. C), represented by circles and colored according to the *p* value for ${\mathscr {C}}$, ${\mathscr {F}}$, ${\mathscr {Q}}$, ${\mathscr {E}}$, $\Delta$, and ${\mathscr {I}}$, respectively.

**Figure 2:**
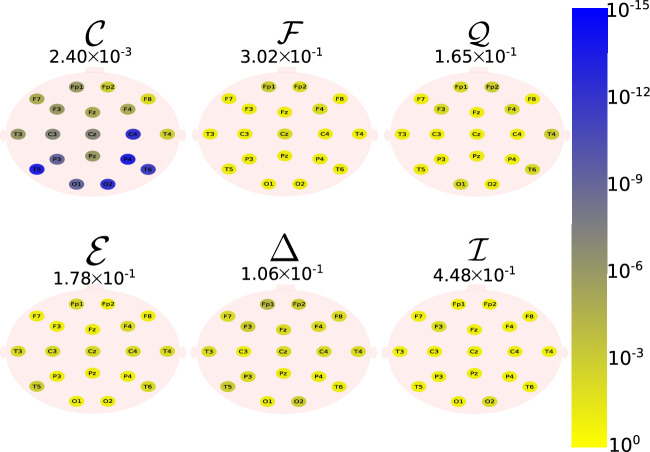
The location on the scalp of the 19 EEG beta band signals (groups A vs. C), represented by circles and colored according to the *p* value for ${\mathscr {C}}$, ${\mathscr {F}}$, ${\mathscr {Q}}$, ${\mathscr {E}}$, $\Delta$, and ${\mathscr {I}}$, respectively.

**Figure 3:**
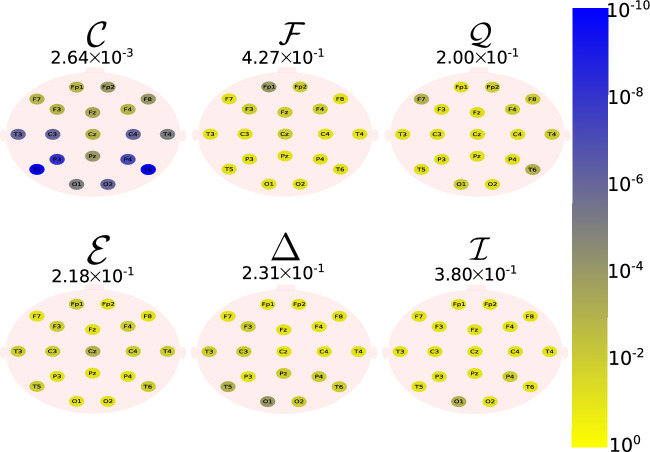
The location on the scalp of the 19 EEG alpha band signals (groups A vs. C), represented by circles and colored according to the *p* value for ${\mathscr {C}}$, ${\mathscr {F}}$, ${\mathscr {Q}}$, ${\mathscr {E}}$, $\Delta$, and ${\mathscr {I}}$, respectively.

**Figure 4:**
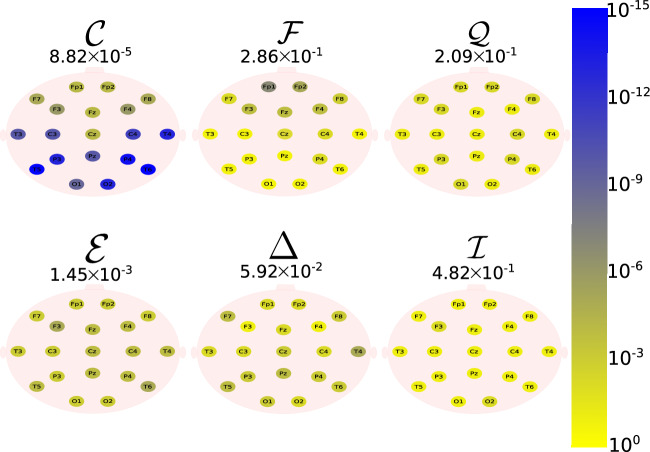
The location on the scalp of the 19 EEG theta band signals (groups A vs. C), represented by circles and colored according to the *p* value for ${\mathscr {C}}$, ${\mathscr {F}}$, ${\mathscr {Q}}$, ${\mathscr {E}}$, $\Delta$, and ${\mathscr {I}}$, respectively.

**Figure 5:**
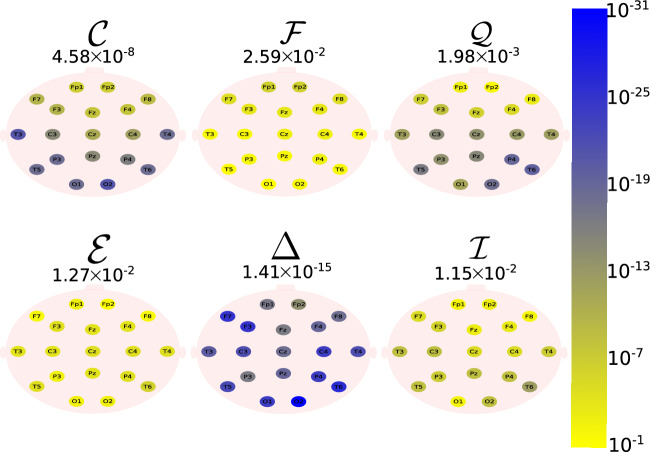
The location on the scalp of the 19 EEG delta band signals (groups A vs. C), represented by circles and colored according to the *p* value for ${\mathscr {C}}$, ${\mathscr {F}}$, ${\mathscr {Q}}$, ${\mathscr {E}}$, $\Delta$, and ${\mathscr {I}}$, respectively.

The Daubechies wavelet filter was used to decompose all the EEG signals in the well-known frequency-bands of the Alzheimer’s neural rhythmic activity^74–79^, i.e., delta (0.5–4 Hz), theta (4–8 Hz), alpha (8–15 Hz), and beta (15–30 Hz). The comparisons were made between the groups A vs C and B vs. D to avoid mixing of eye condition. Figures 1, 2, 3, 4 and -5 show the location of the scalp electrodes for the 19 EEG channels, represented by circles and colored based on the *p* value for ${\mathscr {C}}$, ${\mathscr {F}}$, ${\mathscr {Q}}$, ${\mathscr {E}}$, $\Delta$, and ${\mathscr {I}}$ measures, respectively. For a given measure, the average *p* value over all the electrodes is also displayed in each figure. Darker-colored circles indicate a better distinction between aging and AD.

For the original, non-filtered signals (Fig. 1), $\Delta$ and ${\mathscr {C}}$ produce the best results in distinguishing groups A and C. The worst results were produced by the WE method, with ${\mathscr {E}}=1$. As for the filtered signals, ${\mathscr {C}}$ shows the best discrimination power, when the beta, alpha, and theta frequency bands are considered (Figs. 2, 3, 4), specially in the temporal and occipital lobes. Also, regardless of the feature extraction method, the use of delta waves produces the best differentiation results (Fig. 5). In this case, $\Delta$ followed by ${\mathscr {C}}$ produce the best results, independently of the electrode placement. This result is typically found in AD patients at a later stage of the disease, and corroborates preliminary findings^37^.

**Figure 6:**
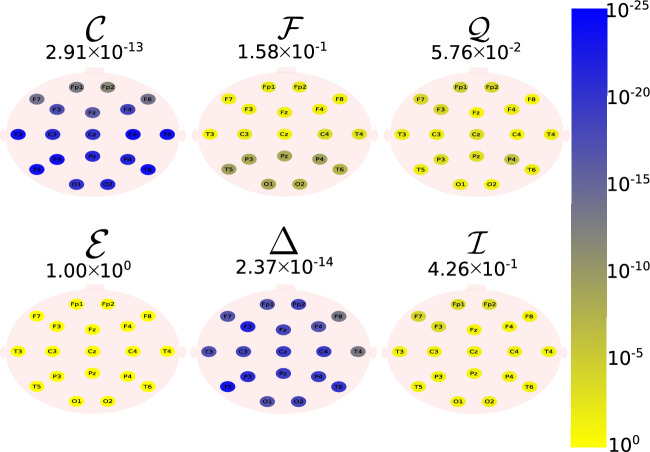
The location on the scalp of the 19 EEG original signal channels (groups B vs. D), represented by circles and colored according to the *p* value for ${\mathscr {C}}$, ${\mathscr {F}}$, ${\mathscr {Q}}$, ${\mathscr {E}}$, $\Delta$, and ${\mathscr {I}}$, respectively.

**Figure 7:**
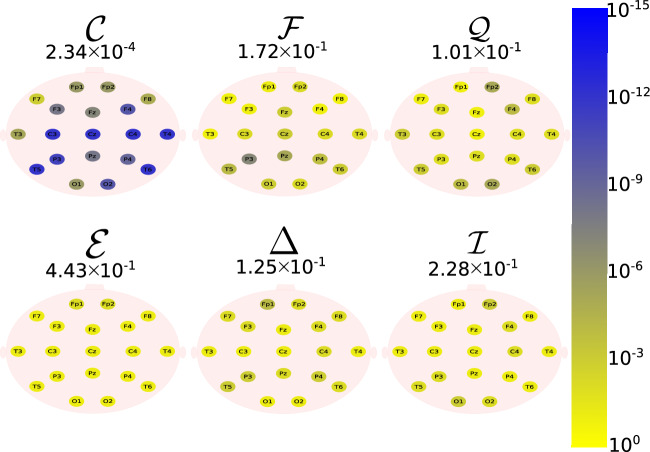
The location on the scalp of the 19 EEG beta band signals (groups B vs. D), represented by circles and colored according to the *p* value for ${\mathscr {C}}$, ${\mathscr {F}}$, ${\mathscr {Q}}$, ${\mathscr {E}}$, $\Delta$, and ${\mathscr {I}}$, respectively.

**Figure 8:**
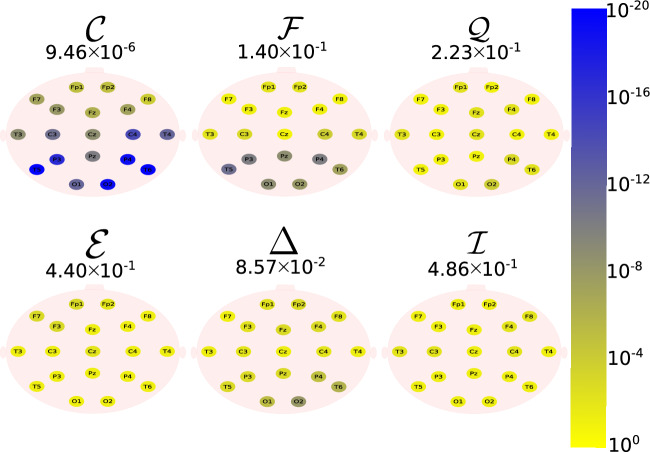
The location on the scalp of the 19 EEG alpha band signals (groups B vs. D), represented by circles and colored according to the *p* value for ${\mathscr {C}}$, ${\mathscr {F}}$, ${\mathscr {Q}}$, ${\mathscr {E}}$, $\Delta$, and ${\mathscr {I}}$, respectively.

**Figure 9:**
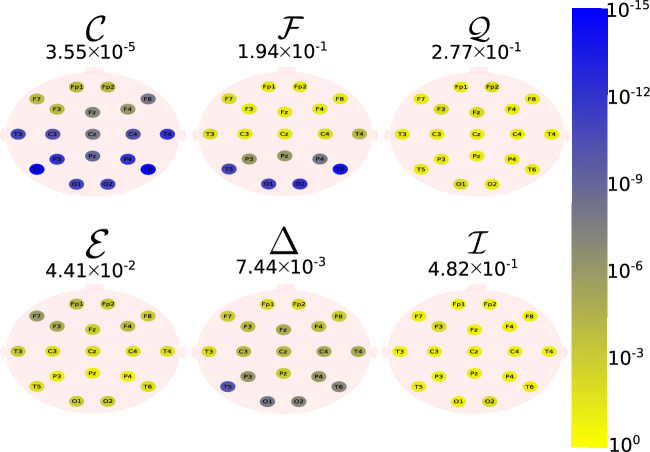
The location on the scalp of the 19 EEG theta band signals (groups B vs. D), represented by circles and colored according to the *p* value for ${\mathscr {C}}$, ${\mathscr {F}}$, ${\mathscr {Q}}$, ${\mathscr {E}}$, $\Delta$, and ${\mathscr {I}}$, respectively.

**Figure 10:**
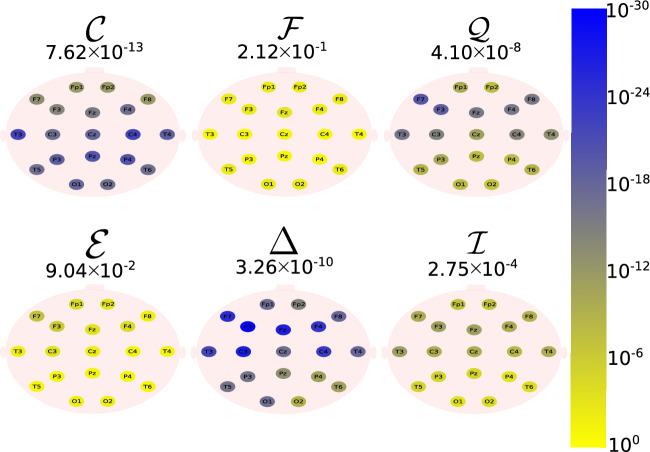
The location on the scalp of the 19 EEG delta band signals (groups B vs. D), represented by circles and colored according to the *p* value for ${\mathscr {C}}$, ${\mathscr {F}}$, ${\mathscr {Q}}$, ${\mathscr {E}}$, $\Delta$, and ${\mathscr {I}}$, respectively.

The same analysis was also performed considering the groups B and D (eyes closed). Figures 6, 7, 8, 9 and 10 depict the location of the scalp electrodes for the 19 EEG channels, which are represented by circles and colored according to the *p* value for the ${\mathscr {C}}$, ${\mathscr {F}}$, ${\mathscr {Q}}$, ${\mathscr {E}}$, $\Delta$, and ${\mathscr {I}}$ measures, respectively. For a given measure, the average *p* value over all the electrodes is also displayed in each figure. Darker-colored circles indicate a better distinction between aging and AD. For the original, non-filtered signals (Fig. 6), $\Delta$ followed by ${\mathscr {C}}$ produce the best results for distinguishing groups B and D. Again, since ${\mathscr {E}}$ is equal to 1.0, this technique is insensitive to the the original EEG signal. As for the frequency bands, ${\mathscr {C}}$ followed by $\Delta$ produce the best results, for all bands, specially delta (Figs. 7, 8, 9, 10). Overall, the best differentiation between healthy and probable AD patients is obtained using delta waves, for all feature extraction methods, regardless whether the subject’s eyes are open or closed.

**Figure 11:**
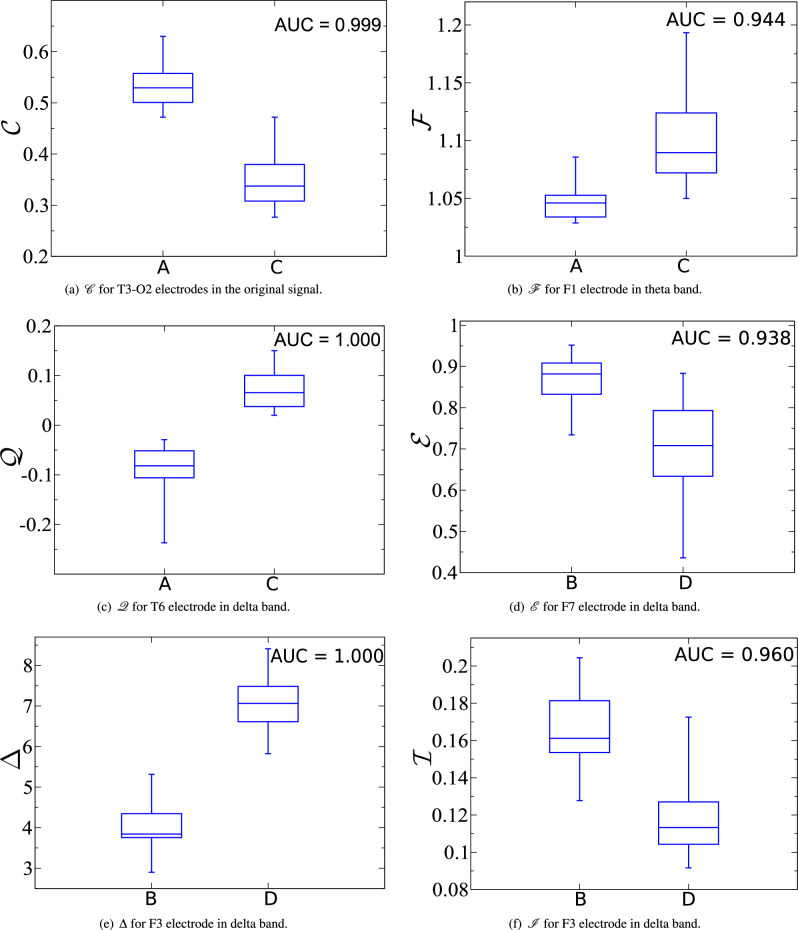
Boxplots for the best electrodes and frequency bands for ${\mathscr {C}}$, ${\mathscr {F}}$, ${\mathscr {Q}}$, ${\mathscr {E}}$, $\Delta$, and ${\mathscr {I}}$, respectively.

Figure 11 presents the boxplots for ${\mathscr {C}}$, ${\mathscr {F}}$, ${\mathscr {Q}}$, ${\mathscr {E}}$, $\Delta$, and ${\mathscr {I}}$, taking into account the electrode placement, the frequency band and the eye condition that best distinguishes healthy from AD patients in each case. Note that ${\mathscr {Q}}$ and $\Delta$ show excellent performance in discriminating patients with different health conditions, with an AUC = 1.0 in both cases. According to some studies^52,56^, the collapse of functional connectivity caused by the loss of neuronal synapses slows the brain’s oscillatory activity, and, thus, the neural activity tends to be less complex. For ${\mathscr {C}}$, ${\mathscr {E}}$, and ${\mathscr {I}}$ this behavior translates into lower values for unhealthy patients; see, respectively, electrodes T3-O2, F7, and F3, in Fig. 11a, d, and f. The opposite trend (unhealthy values higher than healthy ones) is found in ${\mathscr {F}}$, ${\mathscr {Q}}$, and $\Delta$; see, respectively, electrodes F1, T6, and F3, in Fig. 11b, c, and e. Note that our results for ${\mathscr {F}}$ and ${\mathscr {Q}}$ do not agree with^63,80^. In particular, ref.^80^ used the same database of the current study, but a much smaller sample.

**Figure 12:**
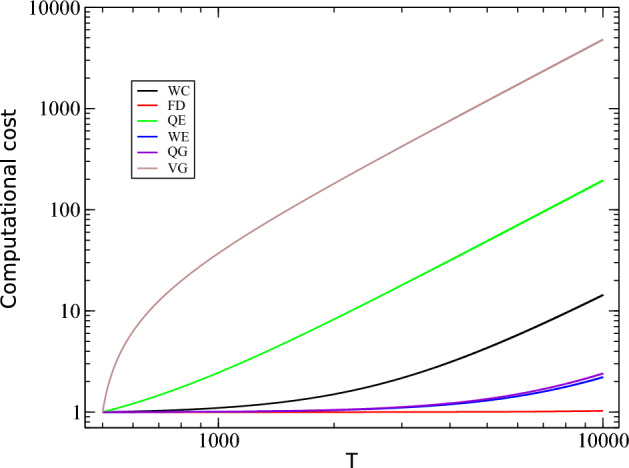
The computational cost for the methods under consideration to computing the measures ${\mathscr {C}}$ (WC), ${\mathscr {F}}$ (FD), ${\mathscr {Q}}$ (QE), ${\mathscr {E}}$ (WE), $\Delta$ (QG), and ${\mathscr {I}}$ (VG) as a function of a random time series (white noite) of length *T*.

Table 1 summarizes the best performance of each technique for the measures ${\mathscr {C}}$, ${\mathscr {F}}$, ${\mathscr {Q}}$, ${\mathscr {E}}$, $\Delta$, and ${\mathscr {I}}$, based on the corresponding *p* values and AUC’s. In each case, the electrode displacement, the frequency band, and the eyes condition were chosen in such a way to best discriminate patients under different health conditions. Although all measures were able to differentiate healthy from AD patients, $\Delta$ displays the best results, regardless the eyes condition (closed or open). Based on the values of the measures ${\mathscr {C}}$, ${\mathscr {F}}$, ${\mathscr {Q}}$, ${\mathscr {E}}$, $\Delta$, and ${\mathscr {I}}$, a support vector machine method was used to individually differentiate healthy elderly subjects from patients with AD. The accuracy (*Acc*), the sensitivity (*Sen*), and the specificity (*Spe*) were calculated (Table 2) using the k-fold cross-validation technique for $K=10$ and the EEG signals under the same conditions established for the previous analysis (Table 1). Although all measures were able to properly classify patients in different health conditions, the values of *Acc* (100%), *Sen* (100%) and *Spe* (100%) reached by $\Delta$, show that this measure is the most efficient one for the classification, regardless of the eye condition. Finally, the computational cost for computing the measures ${\mathscr {C}}$, ${\mathscr {F}}$, ${\mathscr {Q}}$, ${\mathscr {E}}$, $\Delta$, and ${\mathscr {I}}$ was calculated as a function of a random time series (white noise) of length *T*. In each case, the initial and the final time series lengths were $T=500$ and $T=10,000$ points, respectively. In each step, the time series length had an increment of 100 time points, and the computational cost was normalized by the one spent in the initial time step (Fig. 12). Overall, ${\mathscr {F}}$ (FD) followed by ${\mathscr {E}}$ (WE) and $\Delta$ (QG) required the lowest computational effort, while the time spent by the ${\mathscr {I}}$ (VG) method was the highest. This is due to the increasing size of the matrix required to be computed by this method, which reaches $10,000 \times 10,000$ elements in the final step of the simulations.

**Table 1:** The performance of the measures ${\mathscr {C}}$, ${\mathscr {F}}$, ${\mathscr {Q}}$, ${\mathscr {E}}$, $\Delta$, and ${\mathscr {I}}$ that best distinguish healthy from AD patients when electrode displacement, frequency band and eye condition are taken into account.

Measure	Electrode	Band	Groups	*p* value	*AUC*
${\mathscr {C}}$	T3-O2	Original	A vs. C	1.50$\times 10^{-22}$	0.999
${\mathscr {C}}$	T3-C4	Original	B vs. D	3.14$\times 10^{-24}$	0.995
${\mathscr {F}}$	F1	Theta	A vs. C	3.72$\times 10^{-7}$	0.944
${\mathscr {F}}$	T6	Theta	B vs. D	4.70$\times 10^{-14}$	0.906
${\mathscr {Q}}$	T6	Delta	A vs. C	1.08$\times 10^{-20}$	1.000
${\mathscr {Q}}$	F7	Delta	B vs. D	3.71$\times 10^{-20}$	0.980
${\mathscr {E}}$	T6	Delta	A vs. C	2.51$\times 10^{-6}$	0.865
${\mathscr {E}}$	F7	Delta	B vs. D	3.86$\times 10^{-7}$	0.938
$\Delta$	O2	Delta	A vs. C	3.51$\times 10^{-31}$	1.000
$\Delta$	F3	Delta	B vs. D	8.35$\times 10^{-30}$	1.000
${\mathscr {I}}$	T6	Delta	A vs. C	1.06$\times 10^{-12}$	0.958
${\mathscr {I}}$	F3	Delta	B vs. D	1.22$\times 10^{-13}$	0.960

**Table 2:** *Acc*, *Sen* and *Spe* values for the classification of healthy elderly subjects and patients with AD, based on the measures ${\mathscr {C}}$, ${\mathscr {F}}$, ${\mathscr {Q}}$, ${\mathscr {E}}$, $\Delta$, and ${\mathscr {I}}$, and the *K*-fold cross-validation technique.

Measure	Electrode	Band	Groups	$Acc\, (\%)$	$Sen\,(\%)$	$Spe\,(\%)$
${\mathscr {C}}$	T3-O2	Original	A vs. C	97.8	98.7	91.7
${\mathscr {C}}$	T3-C4	Original	B vs. D	97.8	98.7	91.7
${\mathscr {F}}$	F1	Theta	A vs C	95.6	98.7	75.0
${\mathscr {F}}$	T6	Theta	B vs. D	94.6	100.0	58.3
${\mathscr {Q}}$	T6	Delta	A vs. C	97.8	100.0	83.3
${\mathscr {Q}}$	F7	Delta	B vs. D	94.6	98.7	66.7
${\mathscr {E}}$	T6	Delta	A vs. C	88.0	100.0	83.0
${\mathscr {E}}$	F7	Delta	B vs. D	91.3	98.7	41.7
$\Delta$	O2	Delta	A vs. C	98.9	100.0	91.7
$\Delta$	F3	Delta	B vs. D	100.0	100.0	100.0
${\mathscr {I}}$	T6	Delta	A vs. C	90.2	97.5	41.7
${\mathscr {I}}$	F3	Delta	B vs. D	90.2	96.2	50.0

## Discussion

In this paper, the automatic detection of Alzheimer’s disease was performed based on six methods commonly used in the literature. More specifically, for the measures ${\mathscr {C}}$, ${\mathscr {F}}$, ${\mathscr {Q}}$, ${\mathscr {E}}$, $\Delta$, and ${\mathscr {I}}$, according to the corresponding *p* values and AUC’s. Although most of the measures could distinguish between healthy and AD patients (with the exception of ${\mathscr {E}}$), $\Delta$, followed by ${\mathscr {C}}$ display the best results for the original signals, regardless the electrode displacement or the eye condition. In terms of the frequency-bands effect, regardless the method employed, delta waves provide the best differentiation. This finding confirms the prior knowledge that all patients under study may have the disease in its late stage. The values of *Acc* (100%), *Sen* (100%) and *Spe* (100%), obtained by the measure $\Delta$ in combination with the k-fold cross-validation technique, demonstrate that this measure is the most efficient for the classification of individual patients, regardless of their eye condition.

Many research groups have attempted to use computational EEG signal analysis, and many different methods have shown promising results in detecting brain diseases^25–29^. Methods derived from information theory, time-frequency decomposition, and graph theory are examples of computational tools used in distinguishing healthy from unhealthy subjects^30–36^. Several studies in the literature have shown that AD causes slowing in EEG rhythms, reduction in EEG complexity, and changes in synchrony among brain regions^22^. In this sense, the aim of this study was to apply different methods of detecting AD through EEG and investigate the properties of the signals that distinguish the groups of patients. In particular, the capacity for distinguishing AD from healthy elderly subjects was evaluated in terms of the area under the ROC curve and the ANOVA test. In a previous study, it was reported that a high accuracy in detecting AD through EEG was achieved using a small compilation of signals of 24 AD patients and 24 healthy controls^37^. The database in this paper was extended to 160 AD patients, and different computational methods were applied in order to compare their performances in terms of distinguishing groups under different health conditions and in terms of computational cost.

The main goal of the current investigation was to explore the best algorithms capable of differentiating well established AD from normal subjects. Furthermore, our special interest was to evaluate the performance of a new approach known as quantile graphs. It is worth mentioning that according to Rossini^6^, early Alzheimer disease detection can be performed with the used of quantitative EEG with an accuracy of up to $98\%$ percent. In the future, the methodology proposed by Rossini may be used in association with the one described here in order to improve the early diagnosis of this disease through EEG signals.

### Limitations of the study

Most of the computational methods presented here were able to distinguish between healthy individuals and AD patients. However, it is worth mentioning that the subjects under study were not submitted to a definitive pathological diagnosis of AD as well as health controls. As a result, some clinical features of the disease are missing from the database, making it difficult to estimate the efficacy of the methods in providing an early diagnosis for AD patients with only mild cognitive impairment.

## Conclusion

Although early detection is critical to improving the quality of life of AD patients, most of the presently available diagnostic tools, from volumetric magnetic resonance imaging (MRI) to lumbar puncture, are invasive, expensive, and poorly available on community health facilities^6^. In this paper, we applied six non-linear time-series analysis methods to EEG records from 160 AD patients and 24 healthy controls. Our goal was to evaluate the sensibility and robustness of each of the six methods on the task of discriminating AD patients from healthy subjects.

With the exception of the wavelet energy (${\mathscr {E}}$) method, all the other five computational measures were able to distinguish between healthy and AD patients. More specifically, quantile graphs ($\Delta$) followed by wavelet coherence (${\mathscr {C}}$) generated the best results using the original, non-filtered signals, regardless the electrode placement or the eye condition (open or closed). As for the wavelet-filtered signals, the use of delta wave signals improved the discriminating power of all methods, which indicates that the AD patients under study may have the disease in its late stage. Finally, taking into account the electrode placement, the frequency band and the eye condition, and considering only the best results obtained by each method, $\Delta$ showed the best performance in discriminating patients with different health conditions, with $Acc=100\%$, $Sen=100\%$ and $Spe=100\%$ (see Table 2). Finally, taking into account the discrimination performance and the computational cost altogether, $\Delta$ followed by ${\mathscr {C}}$ are the most recommended methods for the AD diagnosis problem.

As for topics for future research, it is necessary to re-evaluate the performance of the methods used in this study when they are applied to a data set that includes patients at different stages of AD, including MCI that later evolves to AD. In addition, the use of low resolution electromagnetic tomography (LORETA), in order to detect the onset of MCI and AD^81^, should also be considered.

## Data Availability

All data used in this study are publicly available at Ref^42^.
